# Hsian-Tsao (*Mesona chinensis* Benth.) Extract Improves the Thermal Tolerance of *Drosophila melanogaster*

**DOI:** 10.3389/fnut.2022.819319

**Published:** 2022-05-09

**Authors:** Yan Huang, Pumo Cai, Xinxin Su, Mingjing Zheng, Wenwen Chi, Shaoling Lin, Zhiwei Huang, Si Qin, Shaoxiao Zeng

**Affiliations:** ^1^College of Food Science, Fujian Agriculture and Forestry University, Fuzhou, China; ^2^College of Tea and Food Science, Wuyi University, Wuyishan, China; ^3^Fujian Provincial Key Laboratory of Quality Science and Processing Technology in Special Starch, Fujian Agriculture and Forestry University, Fuzhou, China; ^4^College of Food Science and Technology, Hunan Agricultural University, Changsha, China; ^5^College of Ocean Food and Biological Engineering, Jimei University, Xiamen, China

**Keywords:** *Mesona chinensis* Benth, *Drosophila melanogaster*, thermal tolerance, antioxidant activities, heat shock protein

## Abstract

Global warming has prompted scientific communities to consider how to alleviate thermal stress in humans and animals. The present study assessed the supplementation of hsian-tsao extract (HTE) on thermal stress in *Drosophila melanogaster* and preliminarily explicated its possible physiological and molecular mechanisms. Our results indicated that the lethal time for 50% of female flies fed on HTE was significantly longer than that of male flies at the same heat stress temperature. Under thermal stress, the survival time of females was remarkably increased in the HTE addition groups compared to the non-addition group. Thermal hardening by acute exposure to 36°C for 30 min (9:00 to 9:30 a.m.) every day could significantly prolong the longevity of females. Without thermal hardening, HTE increased the antioxidant capacity of females under heat stress, accompanied by an increment of catalase (CAT) activity, and the inhibition for hydroxyl radicals (OH⋅) and superoxide anions (⋅O_2_^–^). Superoxide dismutase (SOD) activity and the inhibition for ⋅O_2_^–^ was significantly affected by thermal hardening in the non-HTE addition groups, and significant differences were shown in CAT and SOD activities, and the inhibition for ⋅O_2_^–^ among groups with thermal hardening. After heat exposure, heat shock protein 70 (Hsp70) was only up-regulated in the group with high levels of added HTE compared with the group without and this was similar in the thermal hardening group. It was concluded that the heat stress-relieving ability of HTE might be partly due to the enhancement of enzymatic activities of SOD and CAT, and the inhibition for OH⋅ and ⋅O_2_^–^. However, the expression levels of Hsp70 were not well related to thermal tolerance or heat survival.

## Introduction

As global temperatures rise and climate conditions change, an increasing number of studies have been conducted on the mechanism of animal responses to heat and the alleviation of thermal stress (1). The fruit fly (*Drosophila melanogaster* Meigen) provides a powerful genetic system to investigate the potential mechanisms of temperature adaptation. Thus, this species has widely served as a model organism to examine the contribution of heat shock response to thermal stress (2). Extensive studies have concentrated on the effects of thermal stress on the adaptation of fruit flies such as the population differences in thermal response along the latitude gradient, adaptation to thermal extremes, and evolutionary adaptability in the wild (3–7), and the relationship between heat shock protein 70 (Hsp70) and heat tolerance (8–12). Some attention has been paid to the impacts of dietary manipulations on the thermal tolerance of *D. melanogaster* (13, 14), and also in other animals (1, 15, 16). Andersen et al. (13) discovered that the heat tolerance of fruit flies cultured on a high protein medium was increased compared to the fruit flies fed on a carbohydrate-enriched medium. It was reported by Schriner et al. (14) that *Rosa damascena* extracts compromised the survival of fruit flies in response to heat stress, and it seemed to down-regulate the major heat shock protein Hsp70. Tawfeek et al. (16) found that diets with added antioxidants, especially vitamins and chromium, were vital in overcoming the adverse effects of heat stress on the oxidative status of broilers. These previous studies provide good evidence that diet or nutrition is likely to be a significant factor in the thermal tolerance of fruit flies or other animals.

Hsian-tsao (*Mesona chinensis* Benth.) is an oriental plant resource with both medicinal and edible functions mainly cultivated in Fujian and Guangdong Province of China and Southeast Asian area including India, Indonesia, and Malaysia. In China, hsian-tsao is generally used for producing grass jelly (17) because of its abundant natural colloid, and it also serves as an indispensable raw material in well-known brands of herbal tea, such as Jia Duo Bao and Wong Lo Kat. Related studies on the antioxidant activity of phenolic compounds isolated from hsian-tsao, hsian-tsao gum, and hsian-tsao gum composite films have been conducted (17–20). The alleviation of heat stress in fruit flies by hsian-tsao through antioxidant activity has not been reported. Hsian-tsao has been shown to possess medicinal properties. Jhang et al. (21) confirmed that ethanol extracts from hsian-tsao could inhibit the activity of xanthine oxidase induced by monosodium urate in human acute mononuclear leukemia THP-1 cells. Moreover, it had been reported that aqueous extracts from hsian-tsao had protective effects on the myocardium of streptozotocin-induced diabetic rats (19), resisted acute liver injury induced by tert butyl hydroperoxides, and reduced oxidative stress (22). Additionally, in traditional Chinese medicine, hsian-tsao has the function of “*Xiaoshu*,” which refers to the relief of heat stress and improvement in the resistance to high temperature. So far, the improvement of the body’s thermal tolerance by hsian-tsao has not received sufficient attention.

Heat shock proteins triggered by extreme temperature and other stresses are important components of resistance mechanisms in organisms (23, 24), and function as molecular chaperones to assist in the maintenance of cellular protein structure (2). The fact that members of the Hsp70 family alleviate cellular damage in *D. melanogaster* under heat stress has been recognized for more than two centuries (25–28). In *Drosophila*, several studies have attributed higher levels of Hsp70 to higher levels of thermal tolerance (10, 28–31). Besides Hsp70, thermal stress increases lipid oxidation resulting in the enhancement of reactive oxygen species (ROS) and thus the induction of intracellular oxidative stress (16). Antioxidant enzymes, including catalase (CAT) and superoxide dismutase (SOD), play an important role in protecting cells from the harmful effects of ROS (32). The synthesis of these enzymes is considered to be an important regulator of the stress response in animals. As an endogenous oxidation defense system, the over-expression of SOD and CAT genes increases the longevity of *D. melanogaster* (33–36). Thus, the induction of Hsp70 and the generation of antioxidant enzymes are potential regulatory factors for the improvement of heat tolerance under thermal stress.

To test the hypothesis that hsian-tsao extract (HTE) possesses heat stress-relieving ability, a series of experiments were conducted to explore the effects of HTE on the survival of a model organism (*D. melanogaster*) under thermal stress. The possible physiological and molecular mechanisms of HTE heat stress-relieving ability on *D. melanogaster* were evaluated preliminarily by measuring antioxidant activities and Hsp70 expression levels. This study may provide a reference for the potential HTE-associated thermal tolerance of other organisms.

## Materials and Methods

### Fly Line and Diet

Standard *w*^1118^ flies that originated from the Third Institute of Oceanography, Ministry of Natural Resources (Xiamen, Fujian, China) were used in this study. Colonies had been kept at high numbers of more than 600 for 5 years in the laboratory. Before the experiment, flies were maintained at standard laboratory conditions [25°C, relative humidity (RH) of 50%, and a 12/12 h light/dark cycle] (37), and maintained on a basic medium composed of 34 g cornmeal (Guizhou Taipu Ecological Agriculture Co., Ltd., Guiyang, China), 26 g sucrose (Xiamen Gulong Food Co., Ltd., Xiamen, China), 3.0 g agar (National Pharmaceutical Group Co., Ltd., Beijing, China), 3.0 g yeast (Beijing Biodee Biotechnology Co., Ltd., Beijing, China), and 260 mL of distilled water. Propionic acid of 1.6 mL (Shanghai Macklin Biochemical Co., Ltd., Shanghai, China) was added to the diet to prevent mold growth. Experimental diets were prepared as mentioned above except distilled water was replaced with HTE at different mass concentrations (mentioned in section “Preparation and Ultra-Performance Liquid Chromatography-Mass Spectrometry Analysis of Hsian-Tsao Extract”).

### Preparation and Ultra-Performance Liquid Chromatography-Mass Spectrometry Analysis of Hsian-Tsao Extract

Dried hsian-tsao leaves were provided by Taishan Enterprise Food Co., Ltd (Zhangzhou, Fujian, China). This product was soaked in boiling water at solid–liquid ratios (*w/v*) of 2:1,000, 4:1,000, and 6:1,000 g/mL. The mixtures were boiled with 5% sodium carbonate (National Pharmaceutical Group Co., Ltd.) for 30 min. The infusions were filtered and centrifuged to obtain the supernatant, and HTE at different mass concentrations of 2, 4, and 6 g/L, were obtained.

Ethyl acetate (National Pharmaceutical Group Co., Ltd.) was added to 4 g/L HTE at 1:1 ratio (*v/v*) and then evaporated in a rotary evaporator (Shanghai Yarong Biochemical Instrument Factory, Shanghai, China) at 50°C. A liquid sample of HTE was obtained after redissolution with distilled water. Reference substances (protocatechuic acid, chlorogenic acid, caffeic acid, rutin, ferulic acid, salicylic acid, kaempferol, and scutellarin) (Beijing Solarbio Science & Technology Co., Ltd., Beijing, China) with a total mass of 500 μg were weighed successively, and placed in a 2 mL centrifuge tube and then dissolved in 1 mL of 70% methanol (National Pharmaceutical Group Co., Ltd.) solution. The solution to be measured was obtained. The liquid sample of HTE was separated on an ACQUITY UPLC HSS T3 column (2.1 mm × 100 mm, 1.8 μm, Waters, Quincy, MA, United States). The flow rate was set at 0.3 mL/min and the starting condition for each run was 99:1 mobile phase A (water + 0.5% formic acid): mobile phase B (acetonitrile + 0.5% formic acid) (Shanghai Merck Chemical Technology Co., Ltd.; National Pharmaceutical Group Co., Ltd.) held for 0.25 min, with a ramp to 30:70 (A:B) by 16 min, then to 1:99 by 2 min, and 3 min later reconditioned to initial starting conditions. The peaks were identified according to the retention time of the standards.

### Effects of Various High Temperatures on the Survival of Fruit Flies

Non-mated flies aged 8–10 h were collected and sexed under light ether anesthesia (National Pharmaceutical Group Co., Ltd.). Two blocks contained 24 bottles with 50 flies each (two sexes, four temperature treatments, and three replicates). All flies were provided with a basic medium as food for 3 days under the conditions described above. Next, flies were transferred to experimental medium containing 4 g/L HTE for another 3 days. Afterward, flies were transferred to empty glass bottles and then exposed to 34, 36, 38, and 40°C at 50% RH. The dead flies were counted every 15 min until all were dead, and the lethal time of 50% (LT_50_, the arithmetic mean time of half of the fly deaths that occurred in each group) was determined. Those individuals that were immobile and/or that did not move after shaking test bottles were considered to be dead (38).

### Effects of Hsian-Tsao Extract on the Survival of Female Flies Under Thermal Stress

Female flies aged 8–10 h were collected and randomly divided into 12 bottles with 50 females each (four concentration treatments and three replicates) and reared on a basic medium under the conditions mentioned above. Three days later, females were transferred to medium containing different concentrations of HTE including 0 g/L (control), 2 g/L (low), 4 g/L (medium), and 6 g/L (high), regarded as the CTL, LHTE, MHTE, and HHTE groups, respectively, for another 3 days. Subsequently, females were transferred to the empty bottles and then exposed to a temperature of 36°C and RH of 50% in the incubator (Shanghai Yiheng Scientific Instrument Co., Ltd., Shanghai, China). Deaths were counted every 15 min until all females were dead. Apart from LT_50_, mean survival time (T_*m*_, the weighted average survival time of all flies in each group) and maximum survival time (T_*max*_, the weighted average longevity of fruit flies that died in the last 30 min) were also calculated based on the number of deaths recorded, and the survival curves were plotted. In this study, only female flies were selected for heat tolerance assays and the mechanisms of heat stress-relieving effect by HTE on them, because the survival of female flies under thermal stress markedly exceeded that of males when fed the HTE diet.

### Effects of Hsian-Tsao Extract on the Survival of Female Flies With Thermal Hardening

Female flies aged 8–10 h were collected and divided into 15 bottles with 50 flies each (five treatments and three replicates) under the abovementioned conditions. Three days after rearing, two groups were transferred to basic medium without HTE and the other three groups were moved to medium with HTE of different concentrations including 2 g/L (low), 4 g/L (medium), and 6 g/L (high), respectively, for another 3 days. During this period, one of the groups fed on basic medium and three groups with added HTE all experienced thermal hardening, that is 30-min exposure to 36°C (9:00 to 9:30 a.m. every day) (4) and subsequently returned to the rearing conditions mentioned above. The five groups were the control group without thermal hardening (NTH), the thermal hardening group without HTE addition (TH + NHTE), and HTE groups of different concentrations with thermal hardening (TH + LHTE, TH + MHTE, and TH + HHTE). Subsequently, females of all groups were exposed to 36°C at 50% RH, and deaths were recorded every 15 min until all died. LT_50_, T_*m*_, and T_*max*_ were determined according to the number of deaths recorded, and the survival curves were drawn.

### Gustatory Assay

To rule out that the lifespan extension observed in the survival assay was caused by caloric restriction, gustatory assay was used to examine the food intake of fruit flies (39). Briefly, 180 newly emerged female flies were collected (15 per bottle) and reared on a basic medium for 3 days. Then they were starved for 20 h on Kimwipes paper soaked with distilled water. After that, females were kept on the CTL diet (*n* = 45, 3 bottles), LHTE diet (*n* = 45, 3 bottles), MHTE diet (*n* = 45, 3 bottles), or HHTE diet (*n* = 45, 3 bottles) containing 0.2% sulforhodamine B sodium salt (Acid-red) (Shanghai Yuanye Biotechnology Co., Ltd., Shanghai, China) for 2 h. Food intake of females in the four groups was compared by scoring the degree of abdomen redness with a grading scale ranging from grade 0 (colorless abdomen) to grade 5 (fully red abdomen).

### Antioxidant Activities in Female Flies

Before thermal exposure, female flies were treated according to sections “Effects of Hsian-Tsao Extract on the Survival of Female Flies Under Thermal Stress and Effects of Hsian-Tsao Extract on the Survival of Female Flies With Thermal Hardening,” and then exposed to 36°C and 50% RH for 50 min. Thirty live females were randomly sampled and administered with light ether anesthesia, and then stored in liquid nitrogen (Fuzhou Lianchuang Special Gas Co., Ltd., Fuzhou, China) and kept at −80°C until use. Females from each group were added to 2 mL of normal saline (Sichuan Kelun Pharmaceutical Co., Ltd., Chengdu, China) and homogenized in an ice bath. The homogenates were centrifuged at 4,000 *g* for 10 min at 4°C. The supernatants were extracted for an antioxidant activity assay. Using serum albumin as the standard, the protein concentrations were calculated by the Bradford method (40). A spectrophotometer (Beijing Pullout General Instruments, Beijing, China) was used to determine enzyme activity and antioxidant activity with commercially available assay kits (Nanjing Jiancheng Bioengineering Institute, Nanjing, China) following the manufacturer’s instructions.

#### Catalase Activity

The effect of H_2_O_2_ decomposition on CAT activity was determined spectrophotometrically at 240 nm and CAT activity was calculated (41). One unit of CAT activity was defined as the amount which disintegrated H_2_O_2_ every second per gram of protein. CAT activity was expressed as U⋅g^–1^ protein.

#### Superoxide Dismutase Activity

Superoxide dismutase activity was determined by the xanthine oxidase method. Superoxide anions were produced by the xanthine-xanthine oxidase system and reacted with 2-(4-iodophenyl)-3-(4-nitrophenol-5-phenyltetrazolium chloride) to produce red formazan dye, whose absorbance was determined at 550 nm. SOD can specifically inhibit superoxide anion radicals and decrease the formation of nitrite resulting in reduced absorbance (18). One unit of SOD activity was described as the amount of SOD when the SOD inhibition rate in each milliliter of reaction solution reached 50%, and it was expressed as U⋅mg^–1^ protein.

#### Total Antioxidant Capacity

Many antioxidants in the body can reduce Fe^3+^ to Fe^2+^, and the latter can form a stable complex with phenanthrolines (42). Total antioxidant capacity (T-AOC) was measured at 520 nm by colorimetry.

#### Inhibition Activity for Hydroxyl Radicals

Fenton reaction is the most common chemical reaction to produce OH⋅. The amount of H_2_O_2_ is proportional to the amount of OH⋅ produced by the Fenton reaction. When electron receptors are introduced to the reaction system, a red substance is formed by Griess reagent, and the intensity of the color is proportional to the amount of OH⋅ present. The ability to inhibit OH⋅ was measured in a spectrometer at 550 nm.

#### Inhibition Activity for Superoxide Anions

In this assay, the reaction between xanthine and xanthine oxidase in the organism was used to produce ⋅O_2_^–^. The reaction system became purplish red by adding electron transport substance and Griess reagent. The absorbance of the reaction system could be measured at 550 nm by using a spectrophotometer. When the sample contained ⋅O_2_^–^ inhibitors, the absorbance of the measuring tube was lower than that of the control tube (V_*C*_ as the standard). One unit of activity was defined as the ratio of ⋅O_2_^–^ inhibited per gram of tissue protein reaction for 40 min at 37°C to ⋅O_2_^–^ inhibited by 1 mg V_*C*_ in the reaction system.

### Real-Time PCR

Pre-treatment was conducted as in section “Gustatory Assay.” Live female adults (20 per replicate, 3 replicates per group) were randomly selected under light ether anesthesia, and then stored in liquid nitrogen and kept at −80°C until gene expression of Hsp70 was quantified. Total RNA was extracted using the commercial extraction agent TRIzol [Thermo Fisher Technology (China) Co., Ltd., Shanghai, China]. Females were homogenized in 1,000 μL of TRIzol solution, centrifuged at 12,000 *g* and 4°C for 15 min. Then, the supernatant was extracted and mixed with 200 μL of chloroform (National Pharmaceutical Group Co., Ltd.) for 3 min and oscillated for 10 s before heating for 5 min. The mixtures were then centrifuged at 12,000 *g* and 4°C for 15 min. The upper layer was transferred to a new tube containing isopropanol (National Pharmaceutical Group Co., Ltd.) with equivalent volume. After 20 min of incubation at −20°C, the samples were subjected to centrifugation at 12,000 g and 4°C for 5 min. The sediment was preserved and washed using 1 mL of 75% ethanol (National Pharmaceutical Group Co., Ltd.) followed by recentrifugation. DEPC water [25 μL, Thermo Fisher Technology (China) Co., Ltd.] was added to the RNA pellet and stored at −70°C.

The cDNA was established by a high-capacity cDNA Reverse Transcription Kit [Thermo Fisher Technology (China) Co., Ltd.]. In each reaction, RNA (5 μg) was mixed with 2 μL 10 × RT buffer, 2 μL 10 × random primers, 0.82 μL 25 × dNTP, 1 μL RNase inhibitor, and 1 μL MultiScribe Reverse Transcriptase. The final volume was adjusted to 20 μL with RNase-free water [Thermo Fisher Technology (China) Co., Ltd.]. The cDNA was compounded in the thermocycler GeneAmP PCR system 9700 [(Applied Biosystem, Foster City, CA, United States) and stored at −20°C. Real-time PCR amplification was carried out in a Fast Real-Time PCR system Q5 (Applied Biosystem, Foster City, CA, United States). The target gene Hsp70 (NCBI Reference Sequence NM_169441.2) was normalized with β*-actin* (Table 1), a housekeeping gene used as an internal control (43). Gene expression levels were calculated based on the comparative threshold cycle value and expressed as the ratio of values in the control group before heat exposure.

**TABLE 1:** Primer sequences used in RT-PCR and candidate reference gene analyses in response to heat stress treatments.

Genes		Primer sequences (5′–3′)
**Target genes**		
Hsp70	F	CTTCGATCTGTCCGGCATTC
	R	AGCCGTCCCTTGTCGTTCTT
**Reference genes**		
β*-actin*	F	GGCATCCACGAGACCACCTA
	R	GCGGTGATCTCCTTCTGCAT

### Statistics Analysis

All analyses were performed using Statistical Package for the Social Sciences (SPSS 18.0, SPSS Inc., Chicago, IL, United States). The Kaplan–Meier test was applied to compare the differences between the survival curves. Hsp70 expression data without thermal hardening were transformed using Ln. These data and untransformed data followed a normal distribution and homogeneity of variances based on Kolmogorov-Smirnov and Levene tests. Statistical differences between redness indexes, survival parameters (LT_50_, T_*m*_, and T_*max*_), antioxidant activities (T-AOC, CAT, SOD, and the inhibition for OH⋅ and ⋅O_2_^–^), and Hsp70 relative expressions (without or with thermal hardening) to different levels of HTE concentrations and thermal stress were evaluated using the least significance difference (LSD) test after one-way analyses of variance (ANOVA) for multiple mean comparisons. A general linear model was applied to analyze LT_50_ of fruit flies with different genders under different heat stress temperatures, and a comparison of the differences for LT_50_ between genders and heat stress temperatures was performed using the Duncan test. *P* < 0.05 was considered statistically significant. The figures were drawn using Origin 8.5 (Origin Lab, Northampton, MA, United States).

## Results

### Ultra-Performance Liquid Chromatography-Mass Spectrometry Analysis of Hsian-Tsao Extract

**FIGURE 1:**
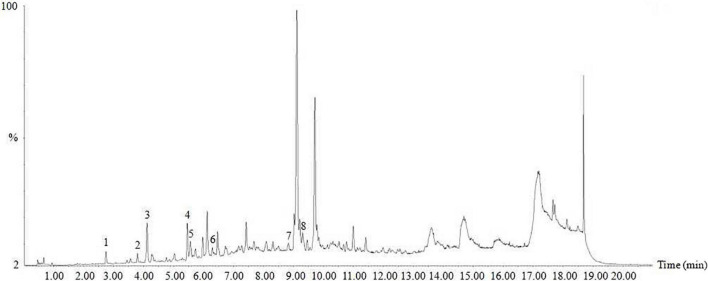
Ultra-performance liquid chromatography-mass spectrometry (UPLC-MS) of hsian-tsao extract (HTE). Peaks: 1, protocatechuic acid; 2, chlorogenic acid; 3, caffeic acid; 4, rutin; 5, ferulic acid; 6, salicylic acid; 7, kaempferol; 8, scutellarin. Other peaks are unknown.

Ultra-performance liquid chromatography-mass spectrometry analysis showed that HTE mainly contained protocatechuic acid (13.7234 μg/g), chlorogenic acid (2.9948 μg/g), caffeic acid (30.0946 μg/g), rutin (14.3868 μg/g), ferulic acid (14.3868 μg/g), salicylic acid (96.4213 μg/g), kaempferol (0.1752 μg/g), and scutellarin (0.1573 μg/g) (Figure 1).

### Effects of Variable High Temperature on the Survival of Fruit Flies

**FIGURE 2:**
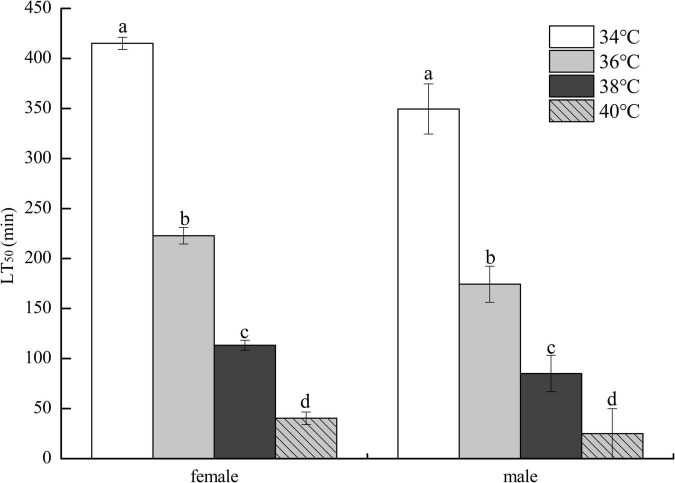
The lethal time of 50% (LT_50_) of fruit flies fed a diet containing hsian-tsao extract of medium mass concentration (4 g/L) under different heat exposure temperatures (34, 36, 38, and 40°C) (150 male or female flies per group, 50 per replicate). Data show mean ± standard deviation (SD). Different lowercase letters indicate significant differences between groups (Duncan test after two-way ANOVA, *P* < 0.05).

As demonstrated in Figure 2, the LT_50_ of HTE-fed female flies decreased as heat stress temperature increased and was significantly longer than that of HTE-fed male flies at the same temperature (*P* < 0.05). Two-way ANOVA showed that the LT_50_ of fruit flies was significantly affected by genders (*P* < 0.01) and heat stress temperatures (*P* < 0.01).

### Effects of Hsian-Tsao Extract on the Survival of Female Flies Under Thermal Stress

**FIGURE 3:**
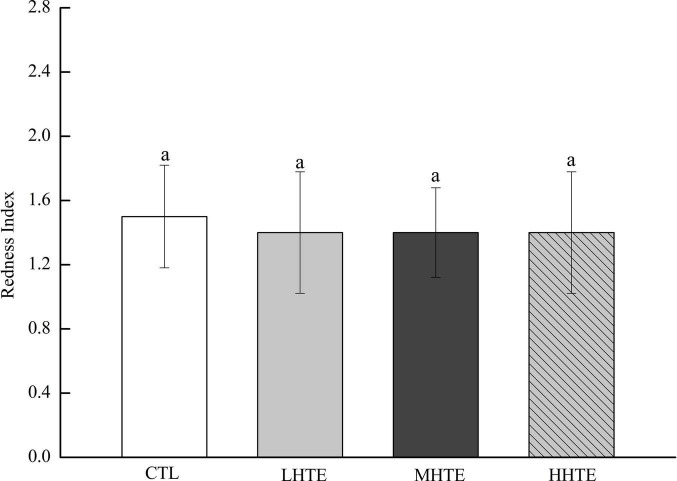
Stomach redness index of fruit flies fed a diet containing hsian-tsao extract (HTE) at low, medium, or high concentrations (LHTE, MHTE, or HHTE) or a non-HTE control diet (CTL) (45 female flies per group, 15 per replicate). Data show mean ± SD. The same lowercase letter indicates no significant differences between groups (LSD test after one-way ANOVA, *P* > 0.05).

**FIGURE 4:**
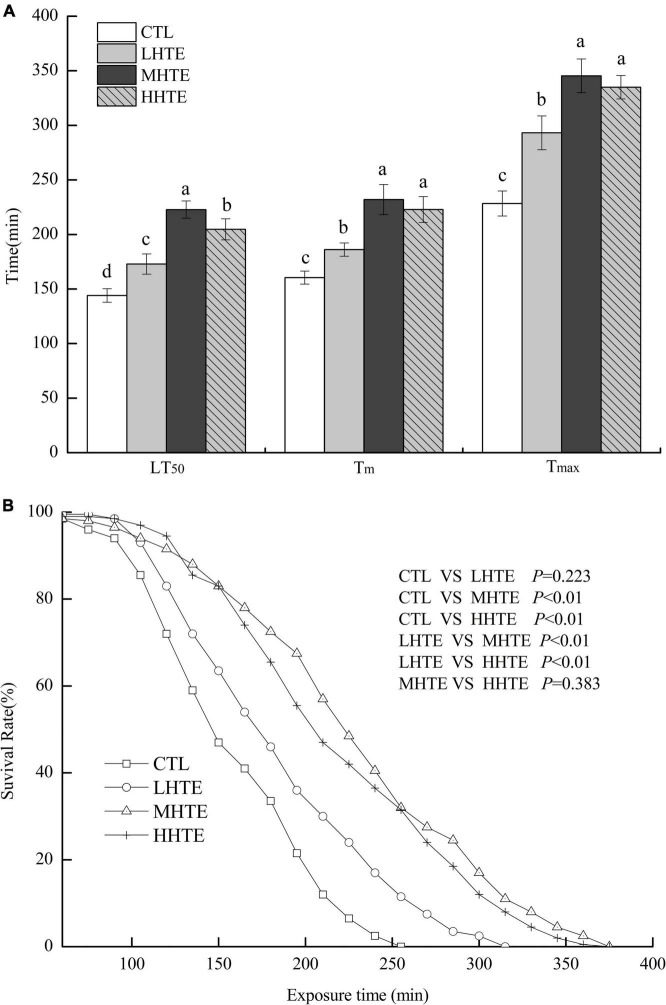
The lethal time of 50% (LT_50_), mean survival time (T_*m*_), and maximum survival time (T_*max*_) **(A)** and survival curves **(B)** of female flies under thermal stress fed a diet containing hsian-tsao extract (HTE) at low, medium, or high concentrations (LHTE, MHTE, or HHTE) or a non-HTE control diet (CTL) (150 female flies per group, 50 per replicate). Data show mean ± SD. Different lowercase letters indicate significant differences between groups (LSD test after one-way ANOVA, *P* < 0.05).

No remarkable differences in food intake of female flies were observed among CTL, LHTE, MHTE, and HHTE groups as reflected by the measurement in the gustatory test (*P* = 0.411, Figure 3). Significant differences in LT_50_ were found among the different treatments (*P* < 0.01), as were in T_*m*_ and T_*max*_ (both *P* < 0.01). Compared with the CTL group, supplementation of HTE significantly lengthened the LT_50_, T_*m*_, and T_*max*_ of females (all *P* < 0.05), and these values all reached a peak in the MHTE group and increased by 54.68, 44.68, and 51.25%, respectively (Figure 4A). There were significant differences in LT_50_ between HTE groups of different mass concentrations (*P* < 0.05), and no differences in T_*m*_ and T_*max*_ were seen between the MHTE and HHTE groups (*P* > 0.05, Figure 4A). The Kaplan–Meier test showed that the addition of MHTE and HHTE significantly affected the survival of female flies compared with the CTL group (both *P* < 0.01), and significant differences were also observed between different HTE groups except for between MHTE and HHTE (both *P* < 0.01, Figure 4B).

### Effects of Hsian-Tsao Extract on the Survival of Female Flies With Thermal Hardening

**FIGURE 5:**
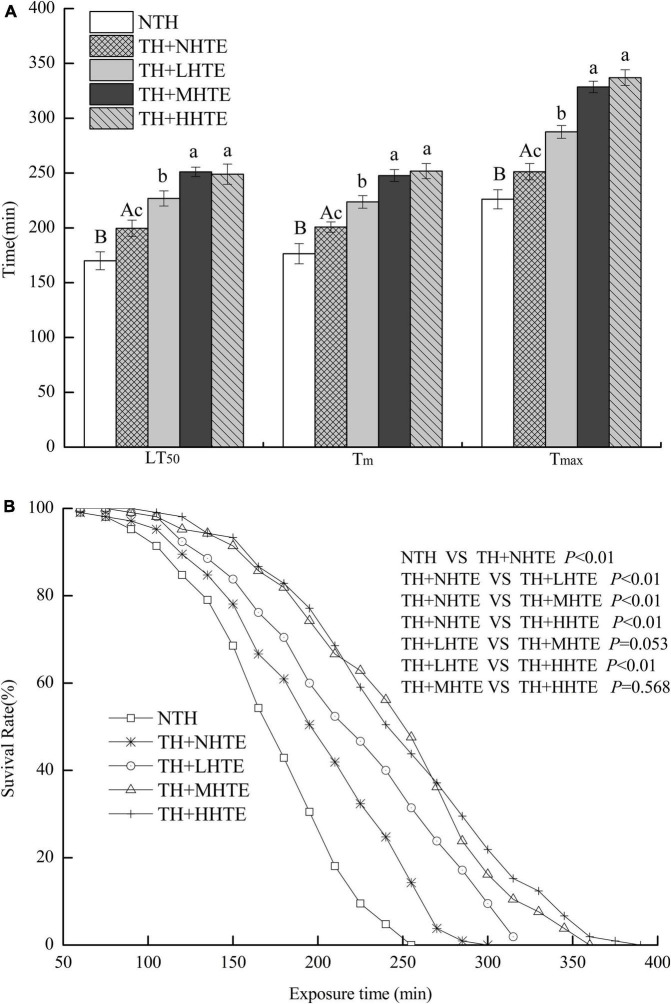
The lethal time of 50% (LT_50_), mean survival time (T_*m*_), and maximum survival time (T_*max*_) **(A)** and survival curves **(B)** of female flies fed a diet containing hsian-tsao extract (HTE) of low, medium, or high concentrations (TH + LHTE, TH + MHTE, or TH + HHTE) with thermal hardening (TH) and a non-HTE control diet with or without TH (TH + NHTE, NTH) (150 female flies per group, 50 per replicate). Data show mean ± SD. Different uppercase letters indicate significant differences between NTH and TH + NHTE, while different lowercase letters indicate significant differences between groups with thermal hardening (LSD test after one-way ANOVA, *P* < 0.05).

The LT_50_, T_*m*_, and T_*max*_ of female flies in the TH + NHTE group were significantly longer than those in the NTH group (all *P* < 0.05), being prolonged by 17.40, 13.77, and 11.11%, respectively (Figure 5A). Females with a pre-exposure to 36°C for 30 min per day demonstrated induced thermal survivorship. One-way ANOVA indicated that survival parameters of females were significantly affected by HTE concentrations (all *P* < 0.01). There were significant differences between TH + NHTE and each HTE group in LT_50_, T_*m*_, and T_*max*_ (all *P* < 0.05). In comparison with the TH + NHTE group, the LT_50_, T_*m*_, and T_*max*_ in the TH + MHTE group were increased by 25.78, 23.42, and 30.75%, respectively (Figure 5A). The Kaplan-Meier test showed that the survival time of females in the TH + NHTE group was highly significantly longer than that of females in the NTH group (*P* < 0.01). Moreover, the addition of HTE markedly increased the survival rate at the same exposure time compared with the TH + NHTE group (all *P* < 0.01), and no remarkable differences were observed between the TH + LHTE and TH + MHTE groups (*P* = 0.053), or the TH + MHTE and TH + HHTE groups (*P* = 0.568, Figure 5B).

### Antioxidant Capacity of Female Flies Under Thermal Stress

#### Antioxidant Capacity of Female Flies Without Thermal Hardening

**FIGURE 6:**
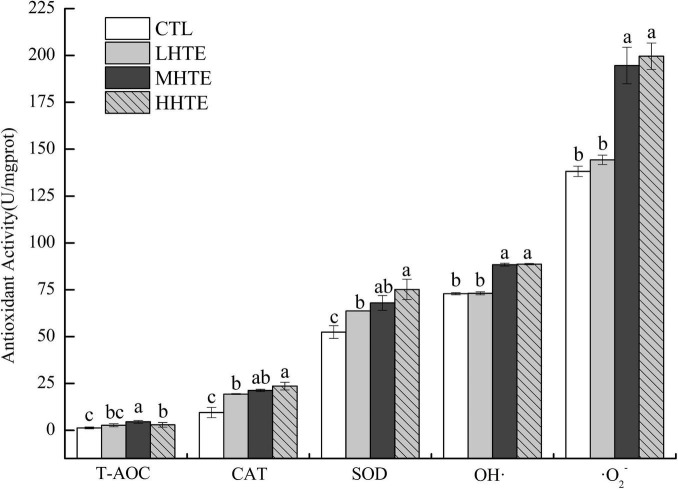
Total antioxidant capacity (T-AOC), catalase (CAT) activity, superoxide dismutase (SOD) activity, and the inhibition for hydroxyl radical (OH⋅) and superoxide anion (⋅O_2_^–^) of female flies under thermal stress fed a diet containing hsian-tsao extract (HTE) of low, medium, or high concentrations (LHTE, MHTE, or HHTE) or a non-HTE control diet (CTL) (90 female flies per group, 30 per replicate). Data show mean ± SD. Different lowercase letters indicate significant differences between groups (LSD test after one-way ANOVA, *P* < 0.05).

Supplementation of HTE could enhance antioxidant activity to some extent. According to one-way ANOVA, CAT activity was significantly affected by HTE concentrations (*P* < 0.05), as was for the inhibition for OH⋅ and ⋅O_2_^–^ (both *P* < 0.01), while no significant differences were observed among groups for T-AOC (*P* = 0.203) and SOD activity (*P* = 0.053). Concerning CAT and SOD activities, remarkable differences were seen between the CTL group and each HTE group (all *P* < 0.05), while for T-AOC, and the inhibition for OH⋅ and ⋅O_2_^–^, a significant increase was observed in the MHTE and HHTE groups compared to the CTL group (all *P* < 0.05, Figure 6).

#### Antioxidant Capacity of Female Flies With Thermal Hardening

Significant differences in SOD activity and the inhibition for ⋅O_2_^–^ were found between the NTH and TH + NHTE groups (both *P* < 0.01). Similar results in CAT and SOD activities, and the inhibition for ⋅O_2_^–^ were found among groups with thermal hardening (all *P* < 0.05). The impacts of HTE concentrations on T-AOC and the inhibition for OH⋅ were not significant (*P* = 0.117 and *P* = 0.228, respectively), as were that of thermal hardening on T-AOC, CAT activity, and the inhibition for OH⋅ (*P* = 0.438, *P* = 0.125, and *P* = 0.191, respectively).

**FIGURE 7:**
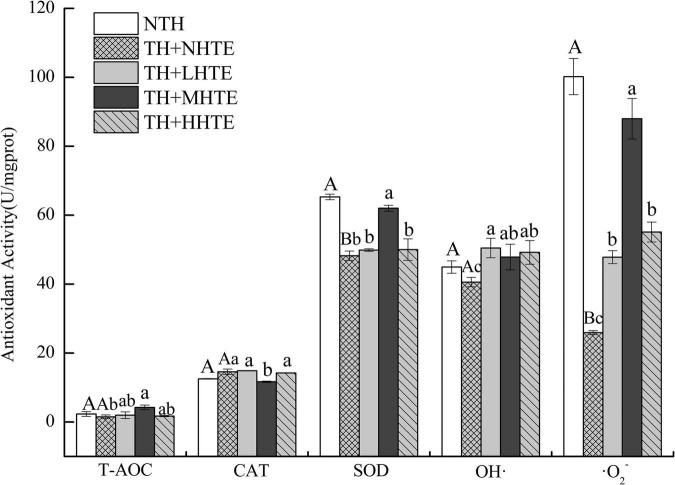
Total antioxidant capacity (T-AOC), catalase (CAT) activity, superoxide dismutase (SOD) activity, and the inhibition for hydroxyl radical (OH⋅) and superoxide anion (⋅O_2_^–^) of female flies under thermal stress fed a diet containing hsian-tsao extract (HTE) of low, medium, or high concentrations (TH + LHTE, TH + MHTE, or TH + HHTE) with thermal hardening (TH) and a non-HTE diet with or without TH (TH + NHTE, NTH) (90 female flies per group, 30 per replicate). Data show mean ± SD. Different uppercase letters indicate significant differences between NTH and TH + NHTE, while different lowercase letters indicate significant differences between groups with thermal hardening (LSD test after one-way ANOVA, *P* < 0.05).

Supplementation of HTE could partially increase the antioxidant activity compared with the TH + NHTE group. SOD activity in the TH + NHTE group was markedly decreased compared with that in the NTH group, and a significant increase in the TH + MHTE group was shown compared with that in the TH + NHTE group (*P* < 0.05). Similar changes were observed in the inhibition for ⋅O_2_^–^, and a remarkable enhancement was seen in all HTE groups in comparison with the TH + NHTE group (all *P* < 0.05), and no significant differences were observed between the TH + LHTE and TH + HHTE groups (*P* = 0.561, Figure 7).

### Gene Expression in Female Flies Under Thermal Stress

#### Influence of Heat Stress on the Gene Expression in Female Flies Without Thermal Hardening

To explore the molecular mechanisms involved in the HTE-promoted survival of female flies under high temperatures, the mRNA levels of gene involved in the relief of heat stress were measured. The relative expression of Hsp70 was affected with a high significance by heat stress (*P* < 0.01) and HTE concentrations (*P* < 0.01). As shown in Table 2, levels of Hsp70 significantly increased when HTE concentrations were enhanced before or after heat exposure (all *P* < 0.05) and were up-regulated dramatically after heat exposure in comparison with before (all *P* < 0.01). Compared with the CTL group, Hsp70 only in the HHTE group was significantly up-regulated after heat exposure (*P* < 0.05), whereas female flies fed on MHTE did not express higher levels of Hsp70 after heat exposure.

**TABLE 2:** The mRNA relative expression levels of heat shock protein 70 (Hsp70) in female flies fed a diet containing low, medium, and high concentrations (LHTE, MHTE, and HHTE, respectively) of hsian-tsao extract (HTE) or a non-HTE control diet (CTL) before and after heat exposure (60 female flies per group, 20 per replicate).

	Hsp70/β-*actin*	
	Before	After
CTL	1.00 ± 0.05^d^	1472667.19 ± 0.25^b^**
LHTE	6.36 ± 0.16^c^	37597.09 ± 0.62^d^**
MHTE	17.59 ± 0.14^b^	263358.17 ± 0.46^c^**
HHTE	89.06 ± 0.45^a^	7029545.58 ± 0.08^a^**

The data of each group were all based on the data of the control group (CTL) before thermal exposure, and the values represent the multiple relationships. Data are expressed as mean ±SD. The data transformed via Ln were analyzed with one-way ANOVA. Asterisks indicate significant differences in Hsp70 expression levels before and after heat exposure (**P < 0.01), whereas different lowercase letters in the same column indicate significant differences between groups (LSD test after one-way ANOVA, P < 0.05).

#### Influence of Heat Stress on the Gene Expression in Female Flies With Thermal Hardening

One-way ANOVA indicated that the impacts of heat stress and HTE concentrations on the relative expression of Hsp70 exhibited highly significant differences (both *P* < 0.01), which was similar to that without thermal hardening. Hsp70 expression levels in all HTE groups were significantly down-regulated compared to that of the TH + NHTE group before heat exposure (all *P* < 0.05). After heat exposure, Hsp70 levels were significantly up-regulated for all groups compared with that before heat exposure (all *P* < 0.01), and there was a drastic improvement for the TH + HHTE group in comparison to the TH + NHTE group (*P* < 0.05, Table 3).

**TABLE 3:** The mRNA relative expression levels of heat shock protein 70 (Hsp70) in female flies with thermal hardening and fed a diet containing low, medium, and high concentrations (TH + LHTE, TH + MHTE, and TH + HHTE, respectively) of hsian-tsao extract (HTE) or a non-HTE control diet (TH + NHTE) before and after heat exposure (60 female flies per group, 20 per replicate).

	Hsp70/β-*actin*	
	Before	After
TH + NHTE	1.00 ± 0.13^a^	141.86 ± 0.29^b^**
TH + LHTE	0.09 ± 0.02^c^	19.09 ± 0.20^c^**
TH + MHTE	0.03 ± 0.01^c^	13.32 ± 0.24^d^**
TH + HHTE	0.74 ± 0.05^b^	272.16 ± 0.63^a^**

The data of each group are all based on the data of the control group (TH + NHTE) before thermal exposure, and the values represent multiple relationships. Data are expressed as mean ± SD. Asterisks indicate significant differences in Hsp70 expression levels before and after heat exposure (**P < 0.01), whereas different lowercase letters in the same column indicate significant differences between groups (LSD test after one-way ANOVA, P < 0.05).

## Discussion

The current study aimed to explore the impact of HTE on the survival of fruit flies under thermal stress. Because antioxidant enzymes, ROS, and heat shock protein expression have potential involvement in the thermal stress response (11, 32, 43, 44), we probed the ability of HTE to regulate these processes.

Our results showed that HTE-fed female flies survived longer than HTE-fed male flies under heat stress temperatures, suggesting that male flies were more sensitive to heat shock than female flies, which was evident from the LT_50_ data (Figure 2). Dahlgaard et al. (45) and Díaz et al. (38) reported similar results for *D. melanogaster* and whitefly *Bemisia tabaci* Gennadius, respectively, and these observations may be caused by the relative expression between constitutive and inducible isoforms of Hsp, representing different functions in flies of different sexes.

Thermal stress induces increased oxidative stress, accompanied by ROS generation, and Hsp expression in animals, and the former can cause oxidative injury such as lipid peroxidation (16, 44, 46, 47). Acting as the first line of defense against oxidation, antioxidant enzyme systems (including SOD, CAT, etc.) play a central role in guarding cells against ROS damage (32, 44), and thus become an important factor for animals in response to heat stress. The current results clearly showed that HTE could prolong the survival time of female flies under heat stress, and the effect was especially pronounced in the MHTE group (Figure 4A). This prolonging effect of HTE was unlikely ascribed to caloric restriction because the food intake was not altered by HTE addition, as reflected by the absence of significant changes in stomach redness index between the CTL and HTE female flies (Figure 3). Treatments that prolong lifespan are often associated with increased resistance to stress (48, 49). CAT activity, and the inhibition for OH⋅ and ⋅O_2_^–^ increased in a concentration-dependent manner, whereas no differences were found between the MHTE and HHTE groups (Figure 6). Increased CAT activity was thought to be a protective response against oxidative stress (50–52), and the enhancement of the inhibition for OH⋅ and ⋅O_2_^–^ may also explain why female flies in the MHTE group had the greatest heat tolerance.

A short-term (minutes or hours) exposure to sub-lethal conditions was generally referred to “hardening” by physiologists (4), and mainly caused reversible physiological changes (53). Heat hardening could prolong the survival time of female flies when challenged with stressful temperatures (Figure 5). In accordance, the thermal tolerance of fruit flies improved after pre-exposure to a sub-lethal temperature (4, 54, 55). Hardening to high temperatures induces oxidative stress or heat shock response, leading to the expression of antioxidants and molecular chaperones (Hsps) (4). Here, we found that the antioxidant indicators including SOD activity and the inhibition for ⋅O_2_^–^in the TH + NHTE group were significantly lowered compared with the NTH group (Figure 7). As an indication of stress response, oxidative stress is usually generated when the organism experiences adverse conditions and indicates the ability of the organism to resist adversity. After thermal hardening, the antioxidant activity of female flies decreased to allow a better adaptation to high temperatures. Additionally, it was clear that some of the antioxidant indexes such as the activities of CAT and SOD, and the inhibition for OH⋅ and ⋅O_2_^–^ in the HTE groups increased compared with those in the TH + NHTE group (Figure 7), suggesting that HTE had the function of enhancing antioxidant activity *in vivo* in female flies. Studies have discovered that the antioxidant activity of HTE may be related to phenolic substances (56, 57). The content of polyphenols in HTE was 57.47 μg/mL by the Folin-Ciocalteu colorimetric method (not measured in the assay). Tea polyphenols of appropriate dose could significantly improve the heat tolerance of fruit flies (unpublished data). Given the results of the previous study, we hypothesized that the phenolic substances in HTE afforded an improved heat tolerance in females. Future work will examine the effects of phenolic substances in hsian-tsao on the survival of flies under heat stress.

In *Drosophila*, exposure to temperatures that evoke stress usually induces Hsp70 synthesis (24, 31, 58). Our results showed that the Hsp70 gene significantly increased in all of the four groups after heat exposure compared with before heat exposure (Table 2). The expression levels of Hsp70 in HTE groups were up-regulated in a concentration-dependent manner before heat exposure, implying that HTE may induce Hsp70 expression before female flies were subjected to heat stress, whereas the Hsp70 gene was only up-regulated in the HHTE group after heat exposure in comparison with the CTL group (Table 2). In this regard, the Hsp70 levels could not explain the data for survival (Figure 4 and Table 2). Also, there were higher levels of Hsp70 in female flies fed on HHTE and basal medium than in female flies with MHTE, which did not correspond to the former being more resistant to heat stress than the latter. This was also consistent with Dahlgaard et al. (45) who discovered that higher levels of Hsp70 in male flies compared with female flies did not present a greater thermal tolerance, suggesting thermal tolerance was not proportional to the levels of Hsp70. With thermal hardening, Hsp70 in each group after heat exposure was up-regulated more than that before heat exposure, and Hsp70 in the TH + HHTE group was up-regulated more in comparison with the TH + NHTE group after heat exposure (Table 3). The same trend was observed in non-thermal hardening treatments (Table 2).

The results above concerning the relationship between Hsp70 expression and thermal tolerance or survival may be ascribed to the following explanations. Hsp70 was involved in enhancing heat tolerance to some extent, but survival under heat shock was complicated and relied on the cumulative stress level (45). As Krebs and Feder (59) mentioned, Hsp expression was not instantaneous, and some time must pass between mild Hsp-inducing stress and intense stress for Hsp expression to promote tolerance. Moreover, Hsp70 was not the only factor influencing thermal tolerance (60, 61), which was indicated by the observation that resistance remained elevated even after the Hsp70 levels had reduced to the basal levels (45). In our study, we merely determined Hsp70 after heat exposure for 50 min at which time female flies were all alive, and Hsp70 in the LHTE and MHTE groups happened to be relatively lower at this point. The survival under heat stress may be controlled by the overall Hsp70 levels during the heat stress process. Hsp70 expression was not examined at different time points after heat exposure, but it did not mean that Hsp70 could not increase at later stages after heat exposure (not measured in the assay). As Dangi et al. (1) observed, the mRNA expression of heat shock proteins was first decreased on short-term heat stress acclimation and then increased on long-term heat stress acclimation, although the animal species and heat exposure patterns were not the same as ours. Acquired heat resistance after hardening seems to be partly ascribed by the up-regulation of the Hsp70 (62, 63). Many studies suggested that Hsp70 seemed to be merely an important part of a molecular cascade, which possibly involved other heat shock proteins being responsible for regulating heat stress resistance in adult *D. melanogaster* (64–69).

In the context of global warming, the frequency of weather with high-temperature extremes has increased significantly. More and more attention has been paid to studies that decipher the response mechanism of animals (especially *D. melanogaster*) under thermal extremes and how to relieve heat stress. Our finding indicating that the HTE diet affects resistance toward heat stress in *D. melanogaster* is of interest to the ecological, evolutional, and physiological fields as well as in nutriology. Moreover, dietary administration is considered a beneficial and economical method to mitigate the adverse effects of thermal stress.

## Conclusion

The current study demonstrated that the incorporation of HTE into the medium was able to prolong the survival time of female flies under heat stress, most likely by the enhancement of CAT activity, and the inhibitory activity for OH⋅ and ⋅O_2_^–^. Thermal hardening could enhance the survival time of female flies under high temperature, along with decreasing antioxidant activity (SOD activity and the inhibition for ⋅O_2_^–^) in the untreated group, and these antioxidant parameters were enhanced partially by the addition of HTE. Additionally, the relationship between Hsp70 expression levels and thermal tolerance or heat survival is still unclear. Therefore, extra efforts will be required to investigate the molecular mechanisms that allow female flies fed with medium containing HTE to survive longer.

## Data Availability Statement

The original contributions presented in the study are included in the article/supplementary material, further inquiries can be directed to the corresponding author.

## Author Contributions

YH, MZ, and WC conceived and designed the experiments. YH, XS, MZ, and WC performed the experiments. YH and MZ analyzed the data. YH wrote the first draft of the manuscript. YH, PC, and SL participated in the revision of the manuscript. ZH, SQ, and SZ helped perform the analysis and with constructive discussions. All authors read and approved the final manuscript.

## Conflict of Interest

The authors declare that the research was conducted in the absence of any commercial or financial relationships that could be construed as a potential conflict of interest.

## Publisher’s Note

All claims expressed in this article are solely those of the authors and do not necessarily represent those of their affiliated organizations, or those of the publisher, the editors and the reviewers. Any product that may be evaluated in this article, or claim that may be made by its manufacturer, is not guaranteed or endorsed by the publisher.
