# Corrigendum: Hsian-Tsao (Mesona chinensis Benth.) extract improves the thermal tolerance of *Drosophila melanogaster*

**DOI:** 10.3389/fnut.2022.953463

**Published:** 2022-09-06

**Authors:** Yan Huang, Pumo Cai, Xinxin Su, Mingjing Zheng, Wenwen Chi, Shaoling Lin, Zhiwei Huang, Si Qin, Shaoxiao Zeng

**Affiliations:** ^1^College of Food Science, Fujian Agriculture and Forestry University, Fuzhou, China; ^2^College of Tea and Food Science, Wuyi University, Wuyishan, China; ^3^Fujian Provincial Key Laboratory of Quality Science and Processing Technology in Special Starch, Fujian Agriculture and Forestry University, Fuzhou, China; ^4^College of Food Science and Technology, Hunan Agricultural University, Changsha, China; ^5^College of Ocean Food and Biological Engineering, Jimei University, Xiamen, China

**Keywords:** *Mesona chinensis* Benth, *Drosophila melanogaster*, thermal tolerance, antioxidant activities, heat shock protein

In the published article, there was an error in Figures 5A, 6 as published. Figure 5A was not the latest version we uploaded, and Figure 6 was wrongly used. The corrected Figures 5A, 6 and their captions appear below.

**Figure 5:**
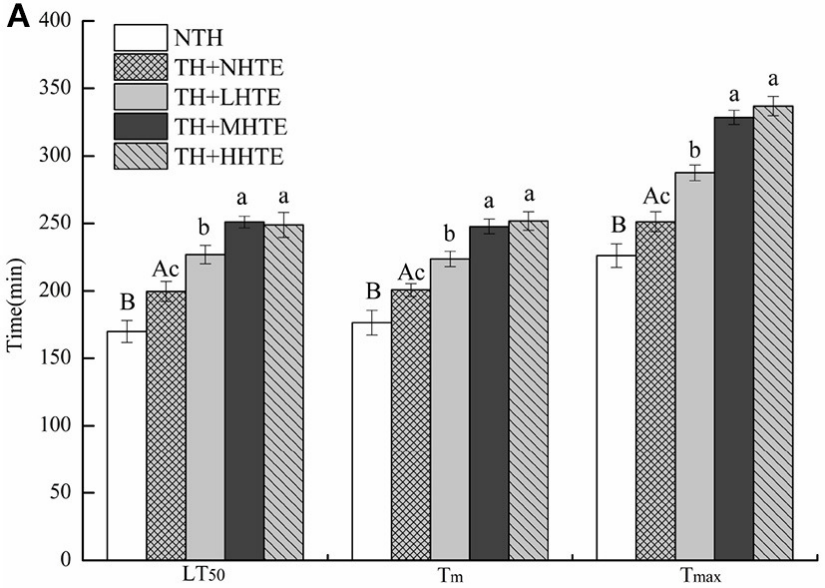
The lethal time of 50% (LT_50_), mean survival time (T_m_), and maximum survival time (T_max_) **(A)** and survival curves **(B)** of female flies fed a diet containing hsian-tsao extract (HTE) of low, medium, or high concentrations (TH + LHTE, TH + MHTE, or TH + HHTE) with thermal hardening (TH) and a non-HTE control diet with or without TH (TH + NHTE, NTH) (150 female flies per group, 50 per replicate). Data show mean ± SD. Different uppercase letters indicate significant differences between NTH and TH + NHTE, while different lowercase letters indicate significant differences between groups with thermal hardening (LSD test after one-way ANOVA, *P* < 0.05).

**Figure 6:**
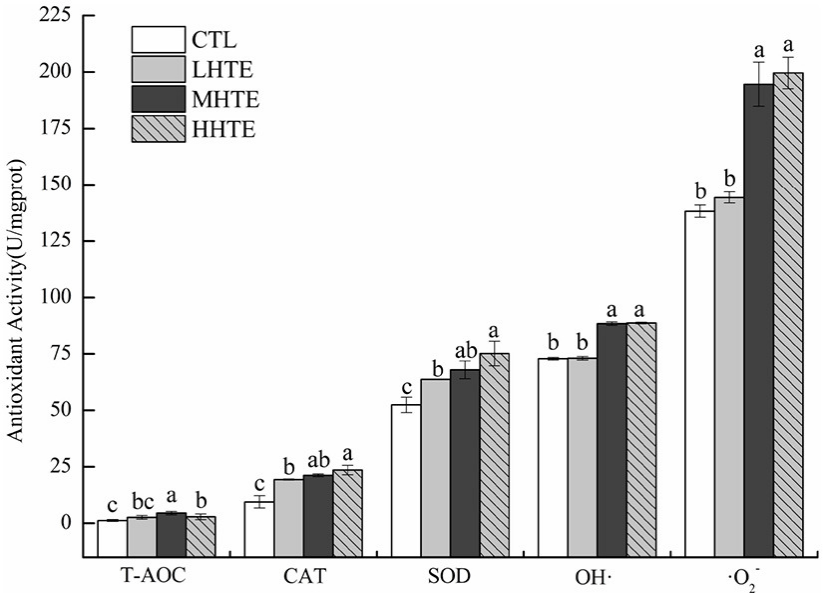
Total antioxidant capacity (T-AOC), catalase (CAT) activity, superoxide dismutase (SOD) activity, and the inhibition for hydroxyl radical (OH·) and superoxide anion (·${\text{O}}_{2^{-}}$) of female flies under thermal stress fed a diet containing hsian-tsao extract (HTE) of low, medium, or high concentrations (LHTE, MHTE, or HHTE) or a non-HTE control diet (CTL) (90 female flies per group, 30 per replicate). Data show mean ± SD. Different lowercase letters indicate significant differences between groups (LSD test after one-way ANOVA, *P* < 0.05).

The authors apologize for this error and state that this does not change the scientific conclusions of the article in any way. The original article has been updated.

## Publisher's note

All claims expressed in this article are solely those of the authors and do not necessarily represent those of their affiliated organizations, or those of the publisher, the editors, and the reviewers. Any product that may be evaluated in this article, or claim that may be made by its manufacturer, is not guaranteed or endorsed by the publisher.

